# Protein Misfolding during Pregnancy: New Approaches to Preeclampsia Diagnostics

**DOI:** 10.3390/ijms20246183

**Published:** 2019-12-07

**Authors:** Elizaveta M. Gerasimova, Sergey A. Fedotov, Daniel V. Kachkin, Elena S. Vashukova, Andrey S. Glotov, Yury O. Chernoff, Aleksandr A. Rubel

**Affiliations:** 1 Laboratory of Amyloid Biology, St. Petersburg State University, 199034 St. Petersburg, Russia; elelovaya@gmail.com (E.M.G.); serg900@yandex.ru (S.A.F.); yury.chernoff@biology.gatech.edu (Y.O.C.); 2 Department of Genetics and Biotechnology, St. Petersburg State University, 199034 St. Petersburg, Russia; 3 Pavlov Institute of Physiology, Russian Academy of Sciences, 199034 St. Petersburg, Russia; 4 Department of Genomic Medicine, D.O. Ott Research Institute of Obstetrics, Gynecology and Reproductology, 199034 St. Petersburg, Russia; vi_lena@list.ru (E.S.V.); anglotov@mail.ru (A.S.G.); 5 Laboratory of Biobanking and Genomic Medicine, Institute of Translation Biomedicine, St. Petersburg State University, 199034 St. Petersburg, Russia; 6 School of Biological Sciences, Georgia Institute of Technology, Atlanta, GA 30332, USA

**Keywords:** preeclampsia, amyloid, protein misfolding, diagnostic, etiology

## Abstract

Preeclampsia (PE) is a multisystem heterogeneous complication of pregnancy remaining a leading cause of maternal and perinatal morbidity and mortality over the world. PE has a large spectrum of clinical features and symptoms, which make diagnosis challenging. Despite a long period of studying, PE etiology is still unclear and there are no reliable rapid tests for early diagnosis of this disease. During the last decade, it was shown that proteins misfolding and aggregation are associated with PE. Several proteins, including amyloid beta peptide, transthyretin, alpha-1 antitrypsin, albumin, IgG k-free light chains, and ceruloplasmin are dysregulated in PE, resulting in toxic deposition of amyloid-like aggregates in the placenta and body fluids. It is also possible that aggregated proteins induce defective trophoblast invasion, placental ischemia, ER stress, and promote PE manifestation. The fact that protein aggregation is an emerging biomarker of PE provides an opportunity to develop new diagnostic approaches based on amyloids special features, such as Congo red (CR) staining and thioflavin T (ThT) enhanced fluorescence.

## 1. Introduction

Preeclampsia (PE) is the human-specific pregnancy complication leading contributor to maternal and fetal mortality worldwide [1]. The clinical symptoms appear after 20 weeks of gestation and include new-onset hypertension, proteinuria, edema, and maternal organ dysfunction [2,3,4]. However, it can be difficult to distinguish PE from other pathologies that are also characterized by hypertension and proteinuria, such as chronic hypertension or glomerulonephropathy.

If left untreated, PE can progress to eclampsia, which is characterized by stroke, seizures, kidney damage, cerebrovascular accidents, microangiopathic hemolytic anemia, liver failure, pulmonary edema—all of these serious consequences of PE can result in maternal death [5,6]. The only effective treatment is the delivery of the placenta and the fetus, leading to iatrogenic prematurity [7]. This is the reason why early diagnosis is so important.

To date, a few promising biomarkers for PE prediction have been found, used alone or in combination [8,9]. They include: a) biochemical markers, such as levels of the placental growth factor (PlGF) [10], of soluble Fms-like tyrosine kinase 1 (sFlt-1) [11,12], and the sFlt-1/PlGF ratio [13], as well as levels of the placental protein 13 (PP13) [14,15], soluble endoglin (sEng) [16,17], pregnancy-associated plasma protein A (PAPP-A) [18], and some others; and b) physiological and biophysical markers, such as mean arterial pressure and uterine artery pulsatility index [9,19]. However, non-invasive and express methods (especially for early diagnosis or prediction of PE), which would not require sophisticated equipment and complex biochemical tests and would allow preventing or starting timely and effective treatment PE before the clinical manifestation of the disease, are still lacking.

Recent studies have shown that proteins misfolding and aggregation is associated with PE. Several proteins, including amyloid beta-peptide, alpha-1 antitrypsin, albumin, IgG k-free light chains, and ceruloplasmin, are dysregulated in PE resulting in deposition of amyloid-like aggregates in the placenta and body fluids. These facts provide an opportunity to develop new diagnostic approaches owing to amyloid have special features.

This review discusses recent findings about proteins misfolding and aggregation during PE and possible diagnostic methods based on these phenomena.

## 2. Diagnosis of PE

Traditionally applied diagnostic criteria of PE are hypertension, appearing after 20 weeks of gestation, combined with proteinuria, that is, the concentration of total protein at the level of 300 mg or higher in a 24 h urine sample [20]. Hypertension is defined as either a systolic blood pressure at the level above 140 mm Hg, or a diastolic blood pressure (BP) at the level higher than 90 mm Hg, as detected at least at two separate occasions. If blood pressure is severe (systolic BP ≥160 and/or diastolic BP ≥110 mm Hg), the measurement should be repeated after 15 min; for less severe blood pressure, repeated measurement should be taken after 3–6 h [4].

However, according to the recommendations of the International Society for the Study of Hypertension in Pregnancy [3], and American College of Obstetricians and Gynecologists [2], proteinuria is not a necessary feature of PE. Rather, PE is diagnosed by the presence of new onset hypertension accompanied by proteinuria and/or renal insufficiency, pulmonary edema, liver involvement, hemolysis, or thrombocytopenia, neurological complications, or fetal growth restriction [21]. The reason for excluding proteinuria as a required criterion is that PE can occur before glomerular capillary endotheliosis becomes severe enough to produce proteinuria [22]. In addition, the standard cut-off for protein concentrations remains uncertain, as the typically used cut-off level of 300 mg per 24 h in urine could be too high [23,24]. Moreover, the urinary dipstick analysis, which is usually used in medical practice, demonstrates a large number of false positive results [25,26], owing to variations in protein excretion, patient diet, and time of urine sampling [24].

Hence, PE is considered a multisystemic disease with non-specific clinical features. The appearance of symptoms such as proteinuria, hypertension, liver failure, and others does not necessarily guarantee a diagnosis of PE. This uncertainty leads to serious issues, as PE requires mandatory delivery. Lack of the rapid, specific, and non-invasive method distinguishing PE from other pregnancy complications and assuring a reliable diagnosis within a short time period is a major challenge for PE research and treatment.

## 3. Etiology and Pathogenesis of PE

Despite numerous studies, the etiology and pathogenesis of PE are still poorly understood. The speed of PE progression is unpredictable, and the subclinical phase is long. This may lead to fetal damage and adaptive clinical manifestations, such as thrombocytopenia, oxidative stress, vascular endothelial dysfunction, systemic inflammation, altered levels of nitric oxide, and aberrant angiogenesis [27]. The central role in the pathogenesis of PE is signified by the observation that the effective treatment for this complication of pregnancy is the early delivery of the fetus and the placenta [28].

In addition, PE is a multifactorial complication of pregnancy including various subclasses. Traditionally, PE is divided into placental or early-onset PE (<34 weeks), and maternal or late-onset PE (≥34 weeks), according to gestational age at diagnosis or delivery [29,30]. These two subtypes seem to have different etiologies. In early-onset PE, abnormal placentation under hypoxic conditions with higher levels of sFlt-1 and lower levels of PlGF take place [31]. Late-onset PE seems to occur from the interaction between a presumably normal placenta and maternal factors and to be a decompensated response to the oxidase stress in the placenta by a dysfunctional maternal endothelium (one aspect of a systemic maternal inflammatory response) [32]. In addition, PE cases can also be divided into subclasses according to their severity [33]. However, these classifications still do not fully reflect the heterogeneity observed in this complication of pregnancy.

Recently, systems biology approaches identified at least three forms of PE based on placental transcriptional phenotyping by using aggregate analysis on previously published PE microarray datasets and clustering the samples based on gene expression [34,35]. The first of these subclasses may arise if the mother demonstrates cardiovascular risk factors resulting in a poor maternal response to pregnancy and development of a later-onset, less severe form of PE (so called “maternal” PE), while the fetus will likely still be normal.

Another “immunological” subclass of PE seems to depend on the presence of immunological risk factors and occur because of incompatibility between the mother and the fetus, which may evoke an immune rejection of the placenta and an immunological presentation of PE.

Finally, the third or “canonical” PE form demonstrates placental dysfunction, elevated expression of known PE markers, and genes associated with poor oxygenation. The traditional view of the pathogenetic mechanisms involved in “canonical” PE is that genetic and environmental factors contribute to the defective deep placentation. Subsequently, the ischemic placenta releases soluble factors into the maternal circulation, which are responsible for the clinical manifestations of the disease [36].

The central hypothesis explaining PE occurrence relates it to the defective trophoblastic invasion with associated uteroplacental hypoperfusion [37]. During normal pregnancy, blood flow in the uterus increases to enable perfusion of the intervillous space of the placenta and to support the growth of the fetus. Physiological transformation of the spiral arteries of the uterus, a process in which trophoblasts invade the uterus and transform the arteries from narrow-diameter to large-diameter vessels, provides the increased blood flow and adequate placenta perfusion [38]. In PE, this remodeling is impaired, the placenta is likely to be deprived of oxygen, which is thought to explain the placental ischemia [39], increased oxidative stress in placenta, and overexpression of soluble fms-like tyrosine kinase-1 (sFlt-1) and soluble Endoglin (sEng) [40,41]. Interestingly, recent systems biology study has shifted the paradigm and shown that maternal inflammation can precede defective trophoblast invasion and shallow placentation [42]. Preexisting maternal diseases or perturbed maternal-fetal-placental immune interactions may be detected in PE earlier and upstream of placental dysfunction, not only downstream, as described before [42].

Several weeks before the appearance of PE clinical manifestations, levels of sFlt-1 and sEng are increased in the serum, and this increase exhibits a positive correlation with the disease severity [40]. sFlt-1 binds to vascular endothelial growth factor (VEGF), which is especially important for maintaining endothelial cell function in the fenestrated endothelium of the brain, liver, and renal glomerulus, and placental growth factor (PlGF), antagonizing their binding to the cell surface. High sFlt-1 and low VEGF/PlGF status contribute to the development of hypertension [43]. A similar effect on VEGF and PlGF is modulated by sVEGFR-1 (a soluble receptor of vascular endothelial growth factors). In recent years, compelling evidence has been collected to support the concept that sVEGFR-1 plays a significant role in the pathogenesis of PE, because of its inhibitory influence on VEGF and PlGF [12,44]. Serum sFlt-1/PlGF ratio has proven to be clinically useful for routine PE diagnosis [10,45,46]. Also, it should be said that automated assays for sFlt-1 and PlGF measurements in serum, plasma, or urine have been already developed [10].

If myometrial segment of the spiral arteries during pregnancy was remodeled deficiently, it can lead to intermittent hypoxia and reoxygenation, which causes oxidative stress [47]. In addition, oxidative stress can occur in a result of the increased placental mitochondrial activity and production of reactive oxygen species (ROS), overwhelming the antioxidation defense mechanisms [48]. Increased levels of ROS, which are usually observed in PE, can lead to lipid peroxidation, protein carboxylation [47], releasing of proinflammatory cytokines and chemokines in blood flow [49], and DNA oxidation. All of these processes promote deterioration of the maternal organism [48].

Placental ischemia in PE is associated with a decreased expression of anti-oxidant heme oxygenase (HO) [50], and this contributes to the increased oxidative stress and the formation of micro-emboli [51]. The HO enzyme exists in two forms, Hmox1, and Hmox2, and converts free heme, which is a source of free radicals, first into biliverdin and then into bilirubin [52]. Hmox is upregulated in hypoxia and ischemia and it produces carbon monoxide, which acts as a vasodilator and decreases perfusion pressure in the placenta [53]. Indeed, increased gene expression of Hmox decreases circulating levels of sFlt-1 [54] and leads to normal pregnancy. Furthermore, trophoblasts express Hmox during pregnancy, and it was shown that inhibition of Hmox results in defective trophoblast invasion in vitro [55]. It has been proposed that pharmacological induction of Hmox expression could relieve hypertension and reduce serum concentrations of sVEGFR-1 and oxidative stress in rodent models [56].

Thus, the imbalance between angiogenic and antiangiogenic factors leads to incomplete spiral artery remodeling, oxidative stress, placental ischemia, and releasing of soluble factors into the maternal bloodstream could contribute to clinical manifestations of PE. This view is confirmed by studies in which the injection of placental extracts of pregnant women with PE into guinea pigs elicited convulsions with liver and kidney involvement, similar to those observed in women with eclampsia [36].

However, the above-mentioned manifestations are not specific only to PE, and errors in physiological remodeling of the spiral arteries per se are not sufficient to cause PE [57], as this failure has also been observed in other obstetric syndromes, such as preterm labor [58], spontaneous abortion, fetal death, and placental abruption [59]. In addition, it should be mentioned that mechanisms responsible for the failure of the physiological transformation of the spiral arteries are not fully understood [36].

By using mRNA fingerprinting, increased levels of neurokinin B (NkB) were identified as the promising marker (and potential causative agent) associated with PE [60]. Indeed, significantly higher levels of NkB in the maternal and umbilical cord blood were observed in preeclampsia, compared to pregnancies without complications [61]. The advantage of excess NkB as a biomarker is its specificity, as elevated levels of NkB are not associated with other known hypertensive disorders [62]. It has been suggested that the defective trophoblast invasion observed in PE leads to placental ischemia and the potential release of NkB as a signal for the maternal organism to increase blood flow to the placenta. NkB acts as a dilatator in the vascular system of the placenta [63]. In addition, NkB is considered as an anti-angiogenic factor that inhibits the assembly of the vascular network of endothelial cells and angiogenesis [64]. NkB can also suppress the expression of some proteins, modulate implantation, and involve in the cellular response to hypoxia and oxidative stress [65]. Trophoblast hypoxia has been shown to stimulate the production of several proteins that are known targets of NkB suppression [66]. It is possible that excess NkB inhibits the normal cellular response to hypoxia and thus contributes to the development of PE [67]. Excess NkB could also be linked to additional clinical manifestations of PE such as thrombocytopenia, inflammation, edema, and eventually, eclampsia [65].

Systemic inflammation and overexpression of toll-like receptor 4 [43,68], as well as high levels of the heat shock protein Hsp70 in the serum [69], production of serum autoantibodies to angiotensin II receptor 1 (AT1-AA), and increased sensitivity to the effects of angiotensin II [70] were also linked to PE, however most of these traits are either not sufficiently specific or technically difficult to diagnose.

An attractive concept postulates that endothelial cell activation and/or dysfunction can be a central feature of PE [36] as vasospasm, a condition in which dysfunctional endothelium releases smaller amounts of prostacyclin and nitric oxide compared to normal and cannot induce relaxation on smooth muscle cells, leading to a reduction in the diameter of the cerebral artery lumen, an arterial spasm, tissue ischemia, and necrosis, is a key component of this disorder, and the PE-associated proteinuria could result from the damage to the fenestrated glomerular endothelium. Indeed, levels of E-selectin and vascular cell adhesion protein 1 were higher in patients with PE than in healthy pregnant women [71], however overexpression of E-selectin is also observed in other obstetric syndromes [72].

The role of apolipoprotein E (ApoE) polymorphism in PE was proposed based on PE-like features, such as hypertension, proteinuria, and increased expression of sFlt-1, detected in the ApoE knockout mice [73,74]. Indeed, certain ApoE alleles are associated with dyslipidemia which may contribute to endothelial cell dysfunction. However, attempts to demonstrate a connection between PE and the particular allele combination of the APOR locus have failed thus far [74].

A novel and intriguing theory about PE pathogenesis is that PE is associated with protein misfolding and aggregation. At the very least, recent data implicate the high-ordered fibrous protein aggregates (amyloids) as a biomarker of PE.

## 4. Protein Misfolding and Amyloid Aggregation in PE

### 4.1. Amyloids and Amyloidogenic Diseases

More than 40 human diseases, including such neurodegenerative disorders as Alzheimer’s, Parkinson’s, and Huntington’s diseases, and transmissible spongiform encephalopathies (TSEs), or prion diseases (such as Creutzfeldt-Jakob disease), are caused by protein misfolding, aggregation, and deposition of fibrous protein aggregates (amyloids) in tissues [75,76,77,78,79,80,81,82]. Examples of amyloidogenic proteins include amyloid β peptide (Aβ) and tau in Alzheimer’s disease [83,84,85], α-synuclein in Parkinson’s disease and related disorders [86,87], and prion protein (PrP) in Creutzfeldt-Jakob disease [88]. Amyloids are highly organized non-covalent cross-β protein polymers that could accumulate in the form of fibrils of 7–10 nm in diameter and are highly resistant to anti-protein agents [76]. Mechanisms of amyloid-induced damage are not yet entirely clear (and could be different in different diseases). In many cases, amyloid formation interferes with the normal protein function, although a loss of protein function per se typically is not equivalent to the respective amyloid disease manifestations. Amyloids can immobilize protein of the same sequence, present in non-amyloid form, and thus spread via the process of nucleated polymerization. Transmissible amyloids, termed prions, can even spread between organisms, causing infection diseases such as TSEs. Recent data indicate that many disease-associated amyloids possess prion properties in specific conditions. Amyloid fibrils can be detected via binding to some dyes that recognize cross-β assemblies. Examples of these dyes include a Congo red (CR) [89,90] and thioflavin T (ThT) [91,92,93]. Amyloids can also be detected by some amyloid-specific antibodies [94], and by electron microscopy (EM) [95,96]. Notably, the ability to form an amyloid is controlled by an amyloid protein itself, as confirmed by the observation that amyloids are formed by mammalian amyloidogenic proteins expressed in heterologous systems, such as yeast [97,98].

### 4.2. Amyloids in PE

**Figure 1:**
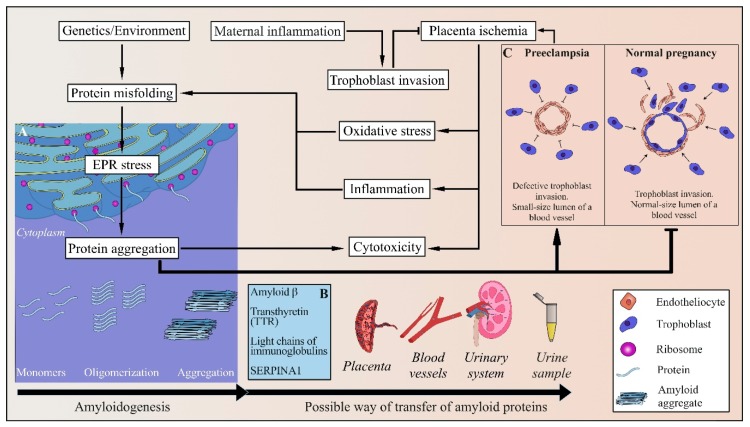
Pathogenesis mechanism of amyloid-based preeclampsia development. In cases of placenta ischemia that is caused by defective trophoblast invasion, oxidative stress, EPR stress, and inflammation may occur. All of these are possible causes of improper protein folding and aggregation. Amyloid aggregation of proteins can cause placenta ischemia. Amyloid aggregates can enter through the placenta into the mother’s blood vessels and enter the urinary system through the bloodstream. Amyloid aggregates are found in the urine of women with preeclampsia. (**A**) During EPR stress, the frequency of protein misfolding increases, which leads to spontaneous aggregation and amyloidogenesis. (**B**) Proteins capable of amyloid aggregation in preeclampsia include amyloid β, transthyretin (TTR), immunoglobulin light chains, and alpha-1 antitrypsin. (**C**) In a normal pregnancy, an invasion of the trophoblasts of the embryo into the spiral arteries of the placenta occurs, which expands the lumen of the vessels and increases the flow of blood to the embryo. During PE, defective trophoblast invasion takes place, which leads to abnormal remodeling of spiral arteries and placenta ischemia.

Recent studies have shown that misfolded proteins accumulate in the urine, serum, and the placenta of women with PE [99,100,101,102,103,104]. Indeed, proteins are vulnerable to misfolding because of changes in genetic and environmental factors [105] (Figure 1). Hence, protein structure can be destabilized under pressures, emerging as a normal part of pregnancy. Recent studies have proven that ischemia, hypoxia, and production of proinflammatory cytokines, associated with PE, can lead to protein misfolding [106] and initiate endoplasmic reticulum (ER) stress [107,108]. Therefore, these conditions may contribute to aggregation and toxic deposition of misfolded proteins in the PE placenta and body fluids. At the same time, it is also possible that aggregated proteins deposited in trophoblasts prevent its normal invasion and induce ischemia and ER stress. It has still to be determined whether or not protein aggregation plays a causative role in PE, triggering the defects in trophoblast invasion, endothelial cell dysfunction, oxidative stress, etc., or just represents a consequence of these aberrations. However, recent data (reviewed below) clearly point to an amyloid as an emerging biomarker of PE.

Since kidney pathology is a hallmark of PE, and proteinuria levels usually correlate with the severity of the disease, Buhimschi et al. [99] performed the proteomic profiling of urine by using mass spectrometry and immunodetection in order to identify biomarkers that would reveal differences between PE patients, healthy women, and, most importantly, patients with proteinuria, which is not related to PE [99]. They found that women with PE (at 34–37 weeks of gestation) display a unique protein profile in their urine that can be used to predict severe PE with high accuracy and makes it possible to distinguish PE from other disorders associated with hypertension and proteinuria during pregnancy [99,101].

By using A11 and polyclonal aAPF antibodies, specifically binding to amyloid-associated epitopes [109,110], the presence of amyloid-type protofibrils and prefibrillar oligomers in the urine samples from women with PE has been detected [101]. These data were confirmed by the detection of fibrillar arborescent conformations tangled together in larger electrodense structures in the PE urine samples using transmission electron microscopy [101]. These structures were similar to images of fibrils extracted from amyloid-laden tissues [111], although the diameter of PE-associated fibrils was somewhat larger. No fibrils were detected in healthy women and patients with chronic hypertension [101].

By using tandem mass spectrometry (MS) and de novo MS-based protein sequencing, the authors identified some isoforms of alpha-1 antitrypsin and albumin as biomarkers in the urine of PE patients [99], that were not present in the urine of non-pregnant women with proteinuria [99]. In addition, the protein component of the urine of pregnant women with PE also contained κ-free light chains of immunoglobulins (IgG), ceruloplasmin, interferon-inducible protein 6-16 (IFI6), and amyloid β [101]. Although it is still unclear which specific protein/s is/are present in the fibrillary form in the PE urine samples, all the above-mentioned proteins, with the exception of IF16, were previously reported to undergo pathologic amyloid or amyloid-like aggregation, and are associated with some known human protein misfolding disorders [112,113]. Therefore, aggregated proteins could be used as a biomarker for predicting the onset of PE.

### 4.3. Alpha-1 Antitrypsin in PE

For example, increased levels of alpha-1 antitrypsin (a serine protease inhibitor, abundantly present in plasma) are detected in patients with diseases associated with an inflammatory component, such as vasculitis, certain infections, etc. Even minor elevation in the levels of serum alpha-1 antitrypsin is accompanied by arterial hypertension [114]. Alpha-1 antitrypsin is fragmented, misfolded, and shown to aggregate in response to oxidative stress [113,115]. Supramolecular aggregates of misfolded alpha-1 antitrypsin, accumulated in hepatocytes and neurons, have recently been identified as factors in the development of serpinopathies, resulting in liver damage and encephalopathy [116]. High frequency of liver pathologies and neurological disorders in PE women agrees with the possibility of alpha-1 antitrypsin misfolding and aggregate accumulation as one of the manifestations of PE [99].

Moreover, in PE, high alpha-1 antitrypsin immunoreactivity was detected not only in urine but also in the serum and placenta, and significant stromal, endothelial, and intravascular deposition of misfolded alpha-1 antitrypsin aggregates has been reported, although the specific pattern of alpha-1 antitrypsin fragmentation present in urine is specific to PE [99].

### 4.4. Light Chains of Immunoglobulins in PE

The aggregation of immunoglobulin light-chains (λ, κ) involved in the pathogenesis of light chain (AL) amyloidosis and multiple myeloma [117,118,119,120]. AL amyloidosis is the most devastating form of systemic amyloidosis [121]. The disease is caused by deposition of amyloid fibrils, constituted by monoclonal immunoglobulin light chains, which are produced by an abnormally proliferative population of plasma cells [117,118,119,120].

Observations that AL amyloidosis most commonly affects kidney, and that patients with PE exhibit proteinuria and kidney damage, suggest that misfolding and aggregation of immunoglobulin light-chains can contribute to the PE-associated pathology.

### 4.5. Amyloid β in PE

Amyloid precursor protein (APP) is a ubiquitously expressed transmembrane glycosylated protein with three major isoforms (APP770, APP751, and APP695), which are produced in the result of alternative splicing of the APP gene. In the normal metabolic pathway, APP is first cleaved by α-secretase to release a soluble N-terminal fragment (sAPPa). Cleaved sAPPa is non-amyloidogenic and functions as a growth factor that promotes cell survival, proliferation, and migration [83,122]. However, in the amyloidogenic pathway, APP is cleaved by β-secretase and then c-secretase releasing the short amyloid β (Aβ) peptide, which is the main component of amyloid plaques observed in the brain in Alzheimer’s disease [122,123,124]. Due to its high propensity for oligomerization and self-assembly, Aβ is considered as a major factor triggering Alzheimer’s disease [124,125,126].

MS analysis has not detected Aβ in the PE urine samples. However, aggregated Aβ could be difficult to detect by MS due to protection from proteolysis, needed for fragmentation that precedes MS analysis. Indeed, APP fragments (including Aβ) were found in the urine of pregnant women with PE by Western blotting [101] with specific ALZ90 monoclonal antibodies [127,128], possible dysregulation of the proteolytic cleavage of APP during PE. This agrees with increased production of α- (ADAM10) and β- (BACE2) in the placenta of women with PE [101]. Expression of one of the γ-secretase genes (*PSEN1*) and of another gene for β-secretase (*BACE1*) was not reported as elevated at the mRNA level in the same tissues, but an increase in the products of these genes in PE trophoblasts has been demonstrated by immunohistochemistry [101]. This discrepancy between approaches is not surprising, as BACE1 expression control includes alternative splicing, post-translational modifications, cellular trafficking, and regulation of degradation [129].

Aside from its presence in the urine, Aβ is accumulated in the placenta of PE women [101]. Previous observations suggested that the placenta expresses APP during normal pregnancy; however, its cleavage and aggregation appear to be increased in the case of PE [104]. It was demonstrated that severe PE is associated with the deposition of amyloid-like aggregates, stained by ALZ90 antibodies [127,128] in the basal plate and villous areas of the placenta [101].

### 4.6. Transthyretin in PE

Recent studies have reported that transthyretin (TTR), a transporter of thyroxine and retinol, which is a known amyloidogenic protein playing a major role in the pathogenesis of familial amyloid polyneuropathy and others TTR-related amyloidosis [130], undergoes dysregulation, misfolding, and aggregation in PE [131]. TTR aggregation may lead to inflammation, oxidative stress, ER stress, and defective deep placentation [131,132]. By using specific ProteoStat dye, specifically binding to aggregated proteins, in combination with ELISA, TTR aggregates were found in the placenta and sera from patients with severe PE [131].

This remains unclear which amyloid protein is primarily responsible for the amyloid aggregation detected in PE urine, and whether or not amyloid formation triggers PE. It is possible that amyloidogenic proteins could induce aggregation of each other, so that several proteins could be present in the amyloid form at the advanced stage of the disease.

### 4.7. Possible Role of the Human Pregnancy Zone Protein

Some secreted proteins, known as extracellular chaperones, such as caseins [133], clusterin [134,135], haptoglobin [136], and alpha-2-macroglobulin (α2M) [136,137], are implicated as inhibitors of protein misfolding and aggregation [133]. Pregnancy zone protein (PZP), which is very similar to α2M, was shown to be significantly elevated in maternal plasma during pregnancy [138]. This can be revealed in serum after 3–4 weeks of gestation [139,140]. It was proposed that the high levels of PZP during pregnancy represent a maternal adaptation counteracting protein aggregation, for example via the formation of stable complexes between PZP and Aβ. PLZ, which is normally present as a dimer in biological fluids, is known to inhibit heat-induced protein aggregation [103] and could be a candidate for the efficient anti-aggregation chaperone similar to dimeric α2M.

Hence, low level of production or dysregulation in the PZP chaperone function resulting in accumulation of misfolded proteins during pregnancy can lead to PE manifestation.

## 5. New Approaches to PE Diagnostics

Over the past twenty years, there has been significant progress in the understanding of pathophysiological mechanisms of PE and in the identification of new potential biomarkers that can be used in the diagnosis of this pregnancy complication. Some efficient approaches, such as serum sFLt1/PlGF ratio [11,141] or sEng measurement in plasma [142], have been introduced. A competing risks model, a Bayes’ theorem based method, which provides an effective approach for the first-trimester prediction of preterm-PE based on maternal characteristics and medical history, biophysical (mean arterial pressure, uterine artery pulsatility index), and biochemical (placental growth factor, soluble fms-like tyrosine kinase-1) markers have been developed [9,19,143]. However, there is still a need for the cheap and specific express approach distinguishing PE and other conditions associated with hypertension and proteinuria.

One such approach could be based on MS-based identification of a unique urine proteomic fingerprint predicting PE. Such a protein profile includes alpha-1 antitrypsin, albumin, IgG k-free light chains, ceruloplasmin, and interferon-inducible protein 6-16 [99], later these data were supplemented by a-1-antitrypsin, complement 3, haptoglobin, and trypstatin [144]. This could be complemented by detection of increased TTR levels in body fluids [71]. A disadvantage of this approach is that MS-based technology is expensive and requires a lot of time and specific equipment not readily available everywhere.

Another set of approaches employs amyloid-binding dyes, such as thioflavin T [72,73,74] and Congo red [89,90]. It was proposed that aggregates of misfolded protein, which are observed in urine, also circulate in the bloodstream and can be detected by using ThT-enhanced fluorescence. However, it was shown that ThT fluorescence in urine and serum was increased only in severe PE but not in mild forms of PE [100]. Another disadvantage of this method is although there were no gestation age-related changes in ThT fluorescence, enhanced ThT fluorescence was shown for late-stage severe PE (29–35 weeks) [100] when the clinical manifestation of PE has already occurred.

**Figure 2:**
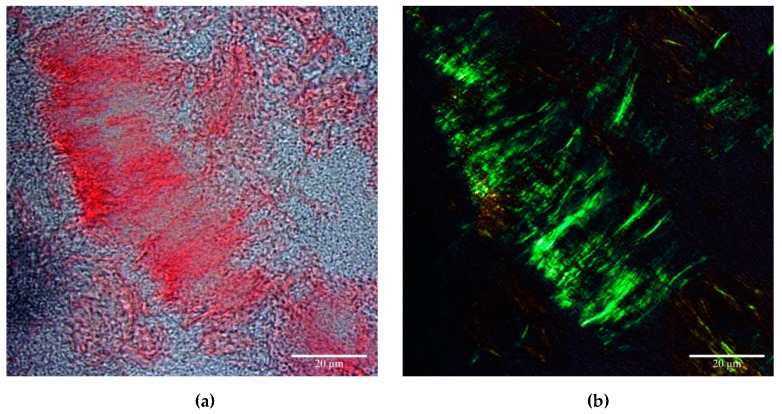
Congo red-stained amyloid aggregates of recombinant *S. cerevisiae* Sup35NM protein. (**a**) Amyloid aggregates of the yeast Sup35NM protein bind to CR; (**b**) CR-stained Sup35NM aggregates demonstrated yellow to apple-green birefringence under polarized light. Data are obtained by D.V. Kachkin.

Buhimschi et al. designed a simpler method based on the fact that women with PE demonstrate urine congophilia—an affinity for the amyloidophilic dye CR [101]. CR staining, followed by birefringence in the polarized light, is the gold standard for the demonstration of amyloids in tissue sections [145] and in vitro (Figure 2). Although the mechanism of CR binding to amyloid fibrils is not fully understood, it is known that this phenomenon is reliant on the affinity of CR for proteins, enriched in β-sheets [90]. Only amyloids (but not any other CR binding compounds) exhibit a phenomenon of birefringence when bound to CR.

**Figure 3:**
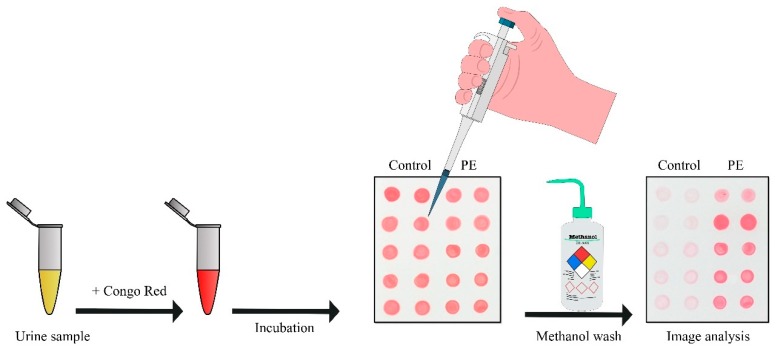
The scheme of the CR dot test for rapid identification of preeclampsia. Urine was mixed with a solution of CR and spotted on a strip of nitrocellulose, which was photographed before and after washing with increasing concentration of methanol. The spots corresponding to PE urine retained the red color, whereas spots of control washed away.

Urinary congophilia (that is, the presence of urea components capable of binding CR) has previously been reported for such a “classic” human prion disease as Creutzfeldt-Jakob disease [146]. Buhimschi et al. have shown that the same approach detects amyloids by CR binding in the urine of women with severe PE. In the case of PE, congophilia develops at an early stage of the asymptomatic phase of PE (more than 10 weeks before clinical manifestation of PE) and progressively develops during pregnancy [101]. The detection approach is employing the absorption of urine proteins on the nitrocellulose filter, followed by staining with CR and washing with methanol (Figure 3). The value of the CR retention after the methanol wash (relative to the value before the wash) was proposed as a diagnostic indicator [101]. Moreover, qualitative (visual) detection based on the presence of the red spots on the filter is also doable.

Later, Rood et al. suggested the Congo Red Dot (CRD) paper test as a simple, univocal, non-invasive clinical tool for rapid PE identification [147]. This modification of the detection approach is based on the fact that CR solution spotted on paper forms hydrogen bonds with cellulose and made a tight circle. However, if in this solution (urine mixed with CR) there are aggregated proteins, they bind to CR and prevent its cellulose binding. Hence, the CR-urine solution spread on the paper forming a wide pink circle. The CRD paper test takes only about 5 minutes and demonstrates high accuracy in PE diagnosis. The authors report that the CRD paper test result can turn positive within 14 days before the clinical manifestation of PE [147]. However, the gestational age of women who took part in the research was generally between 28 and 38 weeks. Usually common PE symptoms can be detected at this stage of pregnancy [20].

Therefore, as of now, diagnostic methods based on protein misfolding during PE are proven to work in the second half of pregnancy, only a few weeks before the PE clinical manifestations. This remains to be determined if these methods are applicable to earlier stages of PE. In the case of amyloid formation playing an important role in disease development, such an applicability is likely, but requires further investigation.

## Abbreviations

PE	Preeclampsia
CR	Congo Red
CRD	Congo Red
BP	Blood Pressure
sFlt-1	Soluble Fms-like tyrosine kinase-1
sEng	soluble Endoglin
PLGF	Placental Growth Factor
sVEGFR	Vascular Endothelial Growth Factor
VEGF	Vascular Endothelial Growth Factor
ROS	Reactive Oxygen Species
HO	Heme Oxygenase
mRNA	messenger Ribonucleic Acid
NkB	Neurokinin B
AT1-AA	Autoantibodies to Angiotensin II receptor 1
Apo E	Apolipoprotein E
TSEs	Transmissible Spongiform Encephalopathies
Aβ	Amyloid β peptide
EM	Electron Microscopy
ER	Endoplasmic Reticulum
TTR	Transthyretin
MS	Mass Spectrometry
igG	immunoglobulins
IFI-6	Interferon-inducible protein 6-16
APP	Amyloid Precursor Protein
sAPPa	soluble N-terminal fragment of APP
α2M	alpha-2-macroglobulin
PZP	Pregnancy Zone Protein
ThT	Thioflavin-T
